# Chorea

**Published:** 1891-01-31

**Authors:** 


### CHOREA.

That the treatment of chorea leaves much to be desired is confessed by all. Fortunately it is a disease which comparatively rarely causes death, so much so that some practitioners, even of many years' experience, have been known to express surprise that a case should ever be so mismanaged as to end fatally, as being contrary to their more fortunate experience. A large proportion of choreic cases in young children are of the milder type from the beginning to the end; it is most unusual for these to take on a very severe character and end fatally. Acute chorea is generally acute from the commencement, and these are the cases which are most eminently unsatisfactory as regards treatment, and so very frequently terminate in death. In the great majority of the milder cases in hospital practice, food and rest are all that is required to cure the patients, and with these a tonic may be often advantageously combined. The adoption of a more definite medicinal course of treatment is, however, undoubtedly of advantage, and arsenic is by almost universal consent the most successful remedy. In his lectures on the "Treatment of Neuroses," Dr. E. C. Sequin states that it is necessary in most cases to go beyond fifteen drops three times a day of Fowler's solution. In some cases when a dose of ten or twelve drops three times a day is reached, gastro-intestinal disturbances and redness of the eyes are apt to appear, and necessitates a leaving off the arsenic for two or three days. After this interval of rest, one can and should begin again at the dose at which one left off, and then continue to increase the amount given, until the really efficacious doses of from eighteen to twenty-five, or even twenty-seven, drops after each meal is reached. He directs that the dose of arsenic should be freely diluted by giving it in a large tumblerful of alkaline water, and should be taken in divided drinks during the hour following the meal. He further insists on the great importance of rest, and that the patient should be kept from other children, and that several members of the family should not be allowed in the room at the same time. The arsenic should be stopped at once, not gradually, as soon as the choreic movements cease. How arsenic acts in chorea we do not know. That it is generally a nerve tonic is, of course, well known, but that it acts especially on the peripheral nerves has been fully proved, at any rate in the lower animals, by the experiments of Dr. Conrad Alexander.

